# Annotations

**Published:** 1911-07-08

**Authors:** 


### Annotations.

### The Annual Grants for Research.

The list of the special researches for which the President of the Local Government Board has authorised payments out of the annual grant voted by Parliament has been announced. The diseases in connection with which the investigations are chiefly concerned appear to be epidemic diarrhœa, plague, and measles. Thus Professor Nuttall, F.R.S. (Cambridge University), is to catalogue the number and kind of fleas found on rats. Dr. J. H. Thursfield (St. Bartholomew's Hospital) will conduct an inquiry into the causes of death in measles. Dr. F. W. Andrewes (St. Bartholomew's) investigates the causes of premature arterial degeneration in man. Dr. C. J. Lewis (Birmingham University) continues his investigation into the prevalence and characteristics of micro-organisms known as non-lactose fermenters in the alimentary canal of infants; and Dr. D. M. Alexander (Liverpool University) is engaged upon the same subject. Dr. Graham Smith (Cambridge University) has two grants: one for inquiry into the incidence of non-lactose fermenters in flies in normal surroundings and in surroundings associated with epidemic diarrhœa, and the other for investigating the possibility of pathogenic micro-organisms being taken up by the larva and subsequently distributed by the fly. Finally, Dr. F. A. Bainbridge (Lister Institute) studies the anaerobic bacteria of the alimentary canal of infants.

### The Attractions of Prison.

While no one would wish to see a reversion to those barbarities and inhumanities of prison life which were swept away finally in the Georgian and early Victorian epochs, still criminologists and prison reformers will do well to take note of the experience of various officials whose close contact with criminals enables them to speak with authority on the mental processes of the average prisoner. Among such officials must be included the coroners; for by a custom at least eight centuries old an inquest must be held on every prisoner who dies during his incarceration. The records of these inquests in modern days show a certain type of prisoner whose frequency is apparently increasing—namely, he (often she) who is committed as a direct or indirect consequence of alcoholic excess, and benefits in health by enforced abstinence; but only to relapse into intemperance and crime after release until a fresh term of imprisonment brings once more enforced teetotalism and renewed health. These prisoners spend much of their time in the hospital wards of the gaol in which they find themselves, and now and then they die there, be it of alcoholic cirrhosis, tuberculosis, pneumonia, or any of the other complaints which may close the career of the habitual drunkard. For these outcasts prison life is so easy and comfortable that there is very little temptation for them to keep in the straight path of hard work and sobriety once they are discharged from gaol. They are the despair alike of judges, gaolers, and of prison gate rescue workers, for their reformation is practically impossible. Yet so soft has public sentimentality rendered the lot of the sick criminal that champagne, chicken, and numerous other luxuries are wasted on these social parasites when they fall ill in prison; in fact, their lot is made a great deal easier than that of many an honest, sober citizen stricken with some deadly disease.

### A Race in the Making.

In a recent article in the *Westminster Review* Mr. J. Liddell Kelly discusses the process of race-fusion which is going on gradually in the Hawaiian Islands, a dependency of the United States. The original Hawaiians are yearly decreasing in numbers, and the author estimates that by the end of this century they will probably be extinct. In their place will be a mixed race in which the Chinese character will predominate, though influenced by admixture with their Polynesian and Caucasian ancestry. Physically and morally the race will be an improvement on the present constituents, though inclined to indolence and probably incapable of the effort necessary for self-government. He is afraid that all hope of making the territory a place for the efforts of a white labouring class must be abandoned. The country will support a few of the better-class whites in comfort, but competition will drive out the farmer and labourer who is not prepared to sink to the level of the native as regards his mode of living.

### The late Sir Rubert Boyce.

The sudden death on June 16 of Sir Rubert Boyce is a serious loss to the profession and to the Liverpool School of Tropical Medicine, which owes so much to his energy and organising abilities. He qualified in 1888 and soon devoted himself to the study of pathology, in which subject he had been for some years Holt Professor at Liverpool. In 1893 appeared his first published study in tropical medicine, a contribution to the ætiology of madura foot. In conjunction with the late Sir Alfred Jones he practically founded the Liverpool School of Tropical Medicine. He himself made expeditions on behalf of the School to Honduras, British Guiana, Barbados, Nigeria, Sierra Leone, and several other colonies; and on yellow fever, to which he especially devoted his attention, Sir R. Boyce had become the first authority in this country. He was one of the first to appreciate the full significance of the researches of Manson, Ross, and the other pioneers of progress in tropical medicine, and to foresee the hidden potentialities—now in the process here and there of being realised—of rendering the tropics habitable for Europeans. He was never afraid to speak his mind; and there is no doubt that attention to hygiene and sanitation was greatly stimulated in some of the West Indies as the result of his visits, and that the public health has benefited greatly as a consequence of his reports.

